# Plastid casein kinase 2 knockout reduces abscisic acid (ABA) sensitivity, thermotolerance, and expression of ABA- and heat-stress-responsive nuclear genes

**DOI:** 10.1093/jxb/eru190

**Published:** 2014-05-06

**Authors:** Yu Wang, Hongping Chang, Shuai Hu, Xiutao Lu, Congying Yuan, Chen Zhang, Ping Wang, Wenjun Xiao, Langtao Xiao, Gang-Ping Xue, Xinhong Guo

**Affiliations:** ^1^State Key Laboratory of Chemo/Biosensing and Chemometrics, College of Biology, Hunan University, Changsha 410082, PR China; ^2^Hunan Provincial Key Laboratory of Phytohormones and Growth Development, Hunan Agricultural University, Changsha, 410128, PR China; ^3^CSIRO Plant Industry, 306 Carmody Road, St Lucia, QLD 4067, Australia

**Keywords:** ABA insensitivity, gene expression, knockout mutation, plastid casein kinase 2, retrograde signalling, thermotolerance.

## Abstract

Plastid casein kinase 2 (CK2) is a major Ser/Thr-specific enzyme for protein phosphorylation in the chloroplast stroma and its kinase activity is regulated by redox signals. To understand the role of CK2 phosphorylation of chloroplast proteins in abiotic stress signalling, an *Arabidopsis* plastid CK2 (*CKA4*) knockout mutant was investigated in terms of the plant response to abscisic acid (ABA) and heat stress. *CKA4* expression was upregulated by ABA and heat treatment. The *cka4* mutant showed reduced sensitivity to ABA during seed germination and seedling growth, and increased stomatal aperture and leaf water loss with a slightly reduced leaf ABA level. The *cka4* mutant was more sensitive to heat stress than the wild-type Columbia-0. The expression levels of a number of genes in the ABA regulatory network were reduced in the *cka4* mutant. Many heat-upregulated genes (heat-shock factors and heat-shock proteins) were also reduced in the *cka4* mutant. The *cka4* mutant showed reduced expression levels of plastid-encoded RNA polymerase target genes (*atpB* and *psbA*). *CKA4* knockout mutation also resulted in a reduction in expression of some critical genes (*PTM*, *ABI4*, and *PRS1*) involved in retrograde signalling from the chloroplast to the nucleus. Similar results were observed in mutant plants with the knockout mutation in both *CKA4* and *CKA3*, which encodes a nuclear CK2 α3 subunit. *CKA3* expression was not responsive to ABA and heat stress. These results suggest that CKA4 is an enhancing factor in abiotic stress signalling through modulating the expression of some molecular players in retrograde signalling.

## Summary

This study demonstrated a role of plastid CK2 in ABA and heat-stress signalling. Its knockout led to reduced ABA sensitivity, thermotolerance, and expression of nuclear genes involved in these processes.

## Introduction

Casein kinase 2 (CK2) is an evolutionary conserved Ser/Thr protein kinase and is generally composed of two different elements (catalytic α-subunit and regulatory β-subunit), which can combine to form a tetramer α_2_β_2_ (Litchfield, 2003; Mulekar and Huq, 2013). The genome of *Arabidopsis thaliana* contains eight genes coding for four α-subunits (αA/CKA1, αB/CKA2, αC/CKA3, and αcp) and four β-subunits (β1/CKB1, β2/CKB2, β3/CKB3, and β4/CKB4) (Salinas *et al.*, 2006). In *Arabidopsis*, three α-subunits (αA/CKA1, αB/CKA2, and αC/CKA3) are located in the nucleus, while αcp contains a chloroplast-targeting sequence motif and is localized exclusively in the chloroplast (Salinas *et al.*, 2006). No β-subunits are present in the chloroplast (Salinas *et al.*, 2006).

CK2 phosphorylates a wide range of proteins (Meggio and Pinna 2003; Mulekar and Huq, 2013). Nuclear CK2 is known to play a role in several important processes in plants, such as cell division and expansion (Moreno-Romero *et al.*, 2008), photomorphogenesis (Hardtke *et al.*, 2000), circadian rhythms (Sugano *et al*., 1998, 1999; Lu *et al.*, 2011), flowering time (Mulekar and Huq, 2012), auxin response (Marquès‐Bueno *et al*., 2011), and abscisic acid (ABA) response (Mulekar *et al.*, 2012).

Involvement of nuclear CK2 in ABA signalling attracts the attention of abiotic stress biologists, as ABA plays a key role in gene regulation in plants under drought and salt stresses (Nakashima and Yamaguchi-Shinozaki, 2013). ABA also regulates various aspects of physiological and developmental processes in plants, such as seed maturation, dormancy and germination, and plant growth (Finkelstein *et al.*, 2002; Nakashima and Yamaguchi-Shinozaki, 2013). Upon perception of drought and salt stresses, ABA levels in plant cells increase, which leads to stomatal closure to reduce leaf water loss and modulates expression of stress-related genes (Fujita *et al.*, 2011). Knockout mutants of CK2 α-subunits in *Arabidopsis* show insensitivity to ABA-induced blockage of seed germination and cotyledon greening (Mulekar *et al.*, 2012), indicating its role in modulating ABA signalling, although the molecular connection of CK2 in ABA signalling is still unknown. To date, the major molecular players in the core ABA signalling network have been identified (Umezawa *et al.*, 2010; Raghavendra *et al.*, 2010; Nakashima and Yamaguchi-Shinozaki, 2013).

Nuclear CK2 is also involved in the heat response in the mammalian system. Heat induces the relocalization of CK2 and activation of its activity (Gerber *et al.*, 2000; Soncin *et al.*, 2003). Mammalian heat-shock factor (HSF) 1 is phosphorylated by CK2, HSF1 activation by heat is correlated with the thermal activation of nuclear CK2, and overexpression of CK2 activates HSF1 activity (Soncin *et al.*, 2003). However, the role of CK2 in modulating the plant heat response is currently unknown. HSF plays a central role in the regulation of heat-shock proteins (HSPs) in all eukaryotic organisms, including plants (Scharf *et al.*, 2012). HSPs are molecular chaperones and are responsible for stabilizing proteins and membranes and assisting in protein refolding under heat stress (Wang *et al.*, 2004). Plants possess basal thermotolerance to cope with temperatures above the optimal for growth and acquired thermotolerance to enhance resistance to otherwise lethally high temperatures. Acquired thermotolerance relies on acclimation to permissive high temperatures, during which time a large number of heat-responsive genes (e.g. HSFs and HSPs) are rapidly induced (Kotak *et al.*, 2007; Wang *et al.*, 2004; Xue *et al.*, 2014).

Studies of chloroplast CK2 have to date focused exclusively on its role in phosphorylation of chloroplast proteins. The chloroplast-targeting CK2 α-subunit is known to be present in other plant species in addition to *Arabidopsis*, named either plastid transcription kinase (PTK) or cpCK2 (Baginsky *et al.*, 1999; Ogrzewalla *et al.*, 2002; Jeong *et al.*, 2004). PTK/cpCK2 is located in the stroma of chloroplasts and is the major kinase for phosphorylation activity in the stroma (Bayer *et al.*, 2012). However, only a few PTK/cpCK2 substrates have been verified experimentally to date, which include sigma factors (SIG1 and SIG6), components of the plastid-encoded RNA polymerase complex (Ogrzewalla *et al.*, 2002; Schweer *et al.*, 2010*b*; Türkeri *et al.*, 2012), RNA-binding proteins (Liere and Link, 1997; Reiland *et al.*, 2009), chloroplast nucleoid-associated protein (MFP1) (Jeong *et al.*, 2004) and the β-subunit of the chloroplast ATPase complex (Kanekatsu *et al.*, 1998). Large-scale phosphoproteome profiling in *Arabidopsis* has shown that potential CK2 phosphorylation sites are over-represented among 174 phosphoproteins in the chloroplast (Reiland *et al.*, 2009). The potential target substrates include carbonic anhydrase, polynucleotide phosphorylase (PNPase), transcriptionally active chromosome (TAC) subunits (TAC10 and TAC16), and a thylakoid associated kinase STATE TRANSITION 7 (STN7) (Reiland *et al.*, 2009). STN7 is known to be involved in retrograde signalling from the chloroplast to the nucleus for regulation of nuclear-encoded photosynthetic genes (Pesaresi *et al.*, 2009). The kinase activities of both PTK/cpCK2 and STN7 are regulated by redox signals in the chloroplast (Pfannschmidt and Liere, 2005; Schönberg and Baginsky 2012). Interestingly, the currently known pathway of STN7 retrograde signalling involves ABA INSENSITIVE4 (ABI4; León *et al.*, 2013); one of its functions is connected to ABA signalling. Recently, retrograde signalling has also been identified to play an important role in heat-mediated induction of HSF and HSP genes, and downregulation of chloroplast ribosomal protein S1 inhibits the activation of HSFA2 and HSP gene expression in *Arabidopsis* during heat stress (Yu *et al.*, 2012). The potential roles of PTK/cpCK2 in phosphorylating STN7 and modulating the expression of plastid genes, which could have a profound effect on nuclear gene expression, indicate that PTK/cpCK2 might have a role in retrograde signalling. Furthermore, redox signalling in the chloroplast, which regulates PTK/cpCK2 kinase activity, is also known to be connected to abiotic stress signalling such as under drought and heat-stress conditions (Sierla *et al.*, 2013; Suzuki *et al.*, 2012).

In this work, we investigated the potential role of *Arabidopsis* plastid CK2 αcp (hereafter named CKA4) in modulating plant responses to ABA and heat stress and regulating nuclear genes during these processes using knockout mutants to provide experimental evidence for its involvement in retrograde signalling. In addition to a *cka4* mutant, two *cka3 cka4* double-knockout mutants (a single T-DNA insertion knocks out both genes) were also used to consolidate observations of the *cka4* mutant phenotypes and to investigate the potential additive role of CKA3, a nuclear CK2 α-subunit, in plant responses to ABA and heat stress. The *cka4* and *cka3 cka4* mutants showed reduced ABA sensitivity phenotypes during seed germination and seedling growth. These ABA-insensitivity phenotypes were supported by attenuation of ABA-mediated upregulation of ABA-responsive genes in the mutants. The knockout mutation of *cka4* and *cka3 cka4* also resulted in reduced thermotolerance and a reduction in expression of heat-responsive HSF and HSP genes during heat stress. No additive role of CKA3 to CKA4 in the *Arabidopsis* response to ABA and heat stress was seen. The knockout mutation of *CKA4* also reduced the expression levels of some target genes of the plastid-encoded RNA polymerase and critical genes involved in known retrograde signalling pathways. These results provide experimental evidence that plastid CK2 is involved in retrograde signalling in plant responses to ABA and heat stress.

## Materials and methods

### Plant materials and growth conditions

*A. thaliana* ecotype Columbia-0 (Col-0) was used as a wild-type control. The *cka4* (CS311135), *cka3-cka4-1* (SALK_022432c), and *cka3-cka4-2* (CS355016) T-DNA insertion mutant lines were obtained from the Arabidopsis Biological Resource Center (http://www.arabidopsis.org). For ABA treatment in a liquid medium, the seedlings were grown initially on Murashige and Skoog (MS) agar plates and were then transferred to a MS liquid medium with or without ABA supplement. MS agar plates were MS medium containing 1% (w/v) sucrose and 0.8% (w/v) agar. Details of ABA and heat treatments and the age of the plants are given in the figure legends. For normal growth conditions, plants were grown in a growth chamber at 22 °C with a 16h light/8h dark cycle. For comparative growth analysis of Col-0 and the mutants, plants were grown in soil under normal growth conditions.

### RNA isolation and expression analysis using quantitative reverse transcription (RT)-PCR

Total RNA was isolated from plants using a Total RNA Purification System kit (Invitrogen). The purified RNA samples were treated with gDNA Eraser (Takara) to remove genomic DNA according to the manufacturer’s instruction. cDNA was synthesized using a PrimeScript^TM^ RT reagent kit (Takara). For quantitative real-time PCR analysis, triplicate PCRs were carried out for each sample in a Mx3000P Real-Time Thermal Cycler (Stratagene). The gene-specific primers used for quantification of mRNA are listed in Supplementary Table S1 at *JXB* online. The *Actin2* gene was used as an internal control. The following amplification procedure was used: 95 °C for 15min, followed by 45 amplification cycles of 95 °C for 15 s, 56 °C for 40 s, and 72°C for 10 s. The specificity of real-time PCR amplification was confirmed by a single peak in melting temperature curve analysis of real-time PCR-amplified products. The quantification of relative mRNA levels was determined as described by Xue *et al.* (2011).

### Verification of T-DNA insertion mutation

**Fig. 1.**
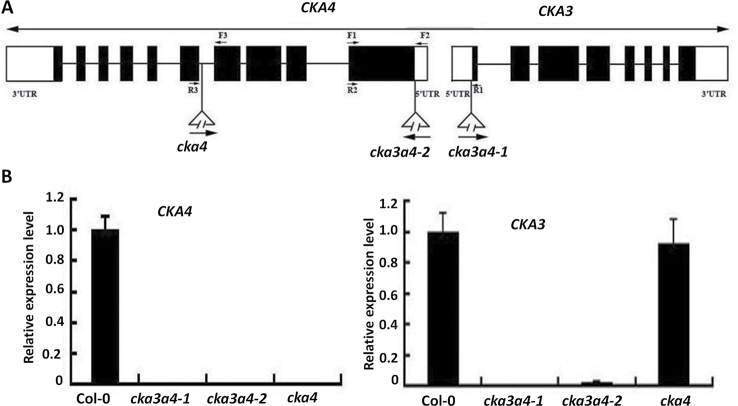
CK2 α4 (*CKA4*) and α3 (*CKA3*) transcript levels in CS311135 (*cka4*), SALK_022432c (*cka3-cka4-1*), and CS355016 (*cka3-cka4-2*) mutants with a T-DNA insertion. (A) Schematic illustration of the *CKA4* and *CKA3* genes and localization of the T-DNA insertion in *cka4* and *cka3 cka4* mutants. Exons and introns are represented by black rectangles and thick lines, respectively. The 5′ and 3′ untranslated regions (UTRs) are depicted as white rectangles. The locations of the T-DNA insertions are indicated by triangles. The positions of PCR primers are indicated by arrows. (B) The *CKA3* and *CKA4* transcript levels in the rosette leaves of 30-d-old Col-0, *cka4*, and *cka3 cka4* mutants and were determined using real-time PCR. Relative expression level was normalized to Col-0, and values are means±standard deviation (SD) of three biological replicates. *Cka3a4-1*, *cka3-cka4-1*; *cka3a4-2*, *cka3-cka4-2*.

First, PCR analysis was used to verify the *cka3 cka4* and *cka4* mutants and to obtain homozygotes of *cka3-cka4-1*, *cka3-cka4-2*, and *cka4*. The positions of primers used for PCR amplification are indicated in Fig. 1A and primers sequences are shown in Supplementary Table S1. Secondly, the expression levels of *CKA3* and *CKA4* were determined in the leaves of 30-d-old plants of the mutants using quantitative real-time PCR analysis and primers designed downstream of T-DNA insertion sites in *CKA4* and *CKA3*. CS31135 obtained from the Arabidopsis Biological Resource Center had a second T-DNA insertion at the At1g66110 gene besides the *CKA4* knockout mutation. To verify that the At1g66110 mutation was absent in the progeny of CS311135, specific primers for At1g66110 downstream of the T-DNA-insertion site (Supplementary Table S1) were used to determine the expression level of the At1g66110 gene. Progeny of CS311135 with the presence of the *cka4* mutation and absence of the At1g66110 knockout mutation were used for this study.

### Germination rate and heat-stress survival rate assays

Three replicate assays, each with at least 300 seeds or seedlings, were conducted. Germination rates were determined 5 d after 0, 0.3, 0.6, or 1 µM ABA treatment in MS agar plates. For analysis of survival rate after heat stress, 2-d-old seedlings were subjected to a heat-stress regime (37 °C for 2h, 22 °C for 2h, and 45 °C for 2h 30min). The survival rates of Col-0 and mutant seedlings were measured after 5 d of recovery at 22 °C.

### Measurements of proline and malondialdehyde (MDA)

After treatment, the samples were frozen in liquid nitrogen and kept at –80 °C until proline and MDA assays were carried out. Free proline concentrations in seedlings were determined using the method described by Bates *et al.* (1973). MDA extraction and measurement were according to Zhang *et al.* (2009).

### Leaf water loss and stomatal aperture measurement

Leaf water-loss analysis was carried out using 30-d-old plants grown in soil. Rosette leaves (≥0.1g) were cut from the wild-type and mutant plants and placed in the growth cabinet at 22 °C with the lights on. Leaf water loss was monitored over a 2h period. Water loss at each time point was presented as the percentage of initial fresh weight.

Stomatal aperture was measured using whole-leaf imaging as described by Pei *et al.* (1998). Rosette leaves of 30-d-old plants were used for preparation of epidermal peels and treated with or without ABA (1 or 10 µM) in a stomatal opening solution (20mM KCl, 1mM CaCl_2_, and 5mM MES/KOH, pH 6.15) for 4h in a growth cabinet. A Nikon TE 2000 inverted fluorescence microscope with ACT-2U software was used to measure stomatal apertures of rosette epidermal peels. The aperture ratios (ratio of stomatal pore width to length) of 40–50 stomata from Col-0 or mutants were determined in each experiment. Three independent experiments were conducted to obtain the mean values of stomatal aperture ratios for each genotype.

### Measurement of ABA levels

The rosette leaves were detached from 30-d-old plants grown in soil and then incubated in liquid MS medium with or without 10 µM ABA for 4h. Tissue ABA levels were measured by liquid chromatography-tandem mass spectrometry (LC-MS/MS 8030 plus; Shimadzu, Japan). A total of 200mg (fresh weight) of ground leaf sample was well mixed with 1ml of 80% methanol in an ultrasonic bath and then kept at 4 °C overnight. After being centrifuged at 15 200*g* for 10min, the supernatant was collected and dried under vacuum. Dried extract was dissolved in 200 µl of 0.1M sodium phosphate solution (pH 7.8). The solution was passed through a Sep-Pak C_18_ cartridge (Waters, USA), followed by elution with 1500 µl of 80% methanol. The eluate was dried under vacuum and then redissolved in 50 µl of 10% methanol; 5 µl of the solution was injected into the LC-MS/MS 8030 system in which an Acquity UPLC BEH column (2.1mm inner diameter×100mm, particle size 1.7 μm; Waters, USA) was used. The column temperature was set at 40 °C and the eluant flow rate was set at 0.25ml min^–1^. The mobile phase comprised solvent A (0.02% acetic acid) and solvent B (acetonitrile) in a gradient mode [time (min)/solvent A (%)/solvent B (%) was 0/90/10, 4/30/70, 5/0/100, and 6/90/10]. The mass spectrometer was set to a multiple reaction monitoring mode using electrospray ionization in a negative ion mode, with a nebulizing gas flow at 3 l min^–1^, a drying gas flow at 15 l min^–1^, a desolvation temperature at 250 °C, and a heat block temperature at 480 °C. ^2^H_6_-ABA (Olchemim, Czech Republic) was used as an internal standard. For ABA, the ionization conditions (pre-bias voltages of 19V for quadrupole 1 and 28V for quadrupole 3, collision energy of 10eV, mass-to-charge ratio of 263:153.2) were employed, while for ^2^H_6_-ABA, the ionization conditions (pre-bias voltages of 20V for quadrupole 1 and 15V for quadrupole 3, collision energy of 11eV, mass-to-charge ratio of 269:/159.2) were employed. ABA concentrations were calculated according to a calibration curve, which was generated by internal standard deuterium-labelled ABA.

### Root and hypocotyl elongation assays

Six-day-old seedlings with a similar shoot length were selected for root-length assays. The seedlings were placed on MS agar plates supplemented with different concentrations of ABA (0, 10, or 40 µM) and grown for 7 d in a growth chamber at 22 °C with a 16h light/8h dark cycle. For heat treatment, the seedlings were subjected to a heat-treatment regime (37 °C for 2h, 22 °C for 2h, and 45 °C for 2.5h) and then grown at 22 °C for 5 d. For dark-growth experiments, 2.5-d dark-grown seedlings were exposed to the above heat-treatment regime and then grown at 22 °C for 5 d in the dark. Root and hypocotyl lengths were measured at the end of the experimental growth periods.

## Results

### CK2 α4 (CKA4) knockout mutant and α3 α4 (CKA3 CKA4) double-knockout mutants

To elucidate the role of CKA4 (At2g23070) in the ABA and heat-stress signalling and the potential additive role of CKA3, we investigated three T-DNA insertion mutants: a *cka4* mutant (CS311135) and two *cka3 cka4* double mutants (SALK_022432c and CS355016). The presence and position of the T-DNA insertion in the progeny of the three mutants were confirmed by PCR using primers flanking the T-DNA insertion sites (data not shown). The T-DNA insertion sites in these three mutants are illustrated in Fig. 1A. *CKA4* is located adjacent to *CKA3* (At2g23080). The two genes run in opposite orientation and part of the coding sequence of one gene is the promoter of the other. Therefore, a T-DNA insertion in SALK_022432c and CS355016 was expected to affect the expression of both *CKA4* and *CKA3*. However, the T-DNA insertion mutation of *CKA4* in CS311135 is unlikely to interfere with the expression of *CKA3*. To determine whether *CKA4* and *CKA3* expression was affected by T-DNA insertion in these mutants, quantitative RT-PCR was performed using primers targeting *CKA4* and *CKA3* transcript sequences downstream of its T-DNA insertion site. As shown in Fig. 1B, neither *CKA4* nor *CKA3* transcripts were detectable in the leaves of SALK_022432c and CS355016. Therefore, these two mutant lines represented *CKA3 CKA4* double-knockout mutants [*cka3-cka4-1* (SALK_022432c) and *cka3-cka4-2* (CS355016)]. In C311135, only the *CKA4* transcript level was not detectable and the expression level of *CKA3* was similar to Col-0 wild-type plants. Therefore, CS311135 is a *cka4* mutant. CS311135 obtained from the *Arabidopsis* Biological Resource Center also has a mutation in At1g66110 encoding a protein belonging to the DUF577 family of unknown function. The At1g66110 mutation was eliminated in the progeny of CS311135, as evidenced by the presence of a similar transcript level of the At1g66110 gene between Col-0 and the CS311135 progeny containing *cka4* (Supplementary Fig. S1 at *JXB* online). The absence of the *CKA4* and *CKA3 CKA4* transcript in the progeny of these T-DNA insertion mutants indicated that they were homozygous.

### A loss-of-function mutation of CKA3 CKA4 or CKA4 leads to reduced ABA sensitivity

**Fig. 2.**
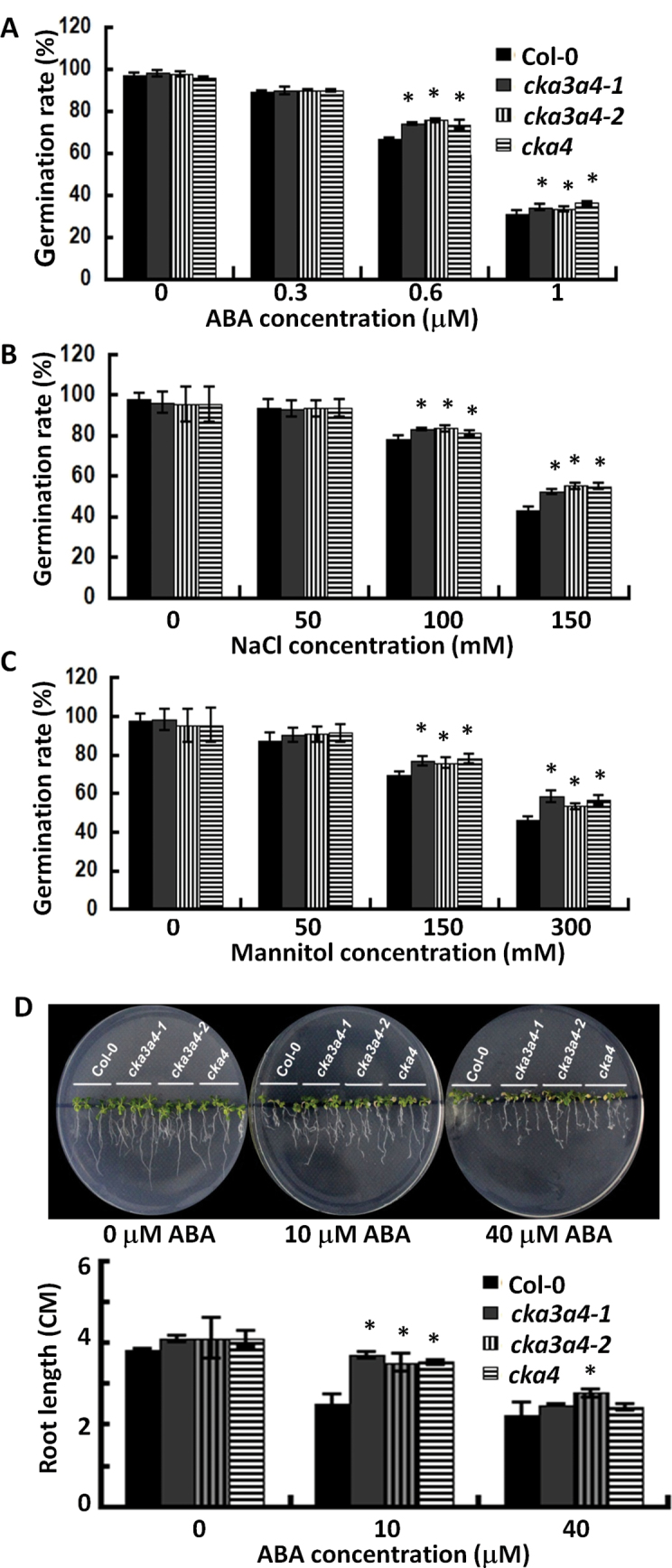
Reduced ABA sensitivity phenotypes of *cka4* and *cka3 cka4* mutants. (A–C) The effects of ABA (A), NaCl (B), and mannitol (C) on seed germination. Col-0 and mutant seeds were germinated on MS agar plates containing 1% sucrose and different concentrations of ABA, NaCl, or mannitol. Radicle emergency rates were determined 5 d after vernalization. Values are means±SD of 300 seeds. (D) Sensitivity of root growth to ABA in Col-0, *cka4*, and *cka3 cka4* mutants. Seedlings of Col-0 and themutants were grown for 6 d on ABA-free medium and then incubated vertically for 7 d on an MS agar plate supplemented with or without ABA. Values are means±SD of 200 seedlings. Asterisks indicate significant differences between mutant and Col-0 (*P<0.05, **P<0.01, using Student’s *t*-test). (This figure is available in colour at *JXB* online.)

ABA is known to regulate seed germination. The efficiency of seed germination in MS agar plates supplemented with various concentrations of ABA was examined by scoring radicle emergence rates 5 d after vernalization. No significant difference in germination between mutants and wild-type Col-0 with 0 and 0.3 µM ABA was observed (Fig. 2A). With ABA concentrations of 0.6 and 1 µM, two *cka3 cka4* double mutants had a higher germination rate than Col-0. This high germination rate was also observed in CS311135, indicating that *CKA4* knockout alone can account for the observed ABA-insensitive phenotype.

The effect of *CKA4* and *CKA3 CKA4* mutation on the germination rate of seeds under salt or osmotic (mannitol) stress was also investigated. Seed germination was carried out in MS agar plates supplemented with NaCl (0, 50, 100, or 150mM) or mannitol (0, 50, 150, or 300mM). It is well established that ABA accumulates in plants under these stresses (Zhu, 2002). Significantly higher germination rates were observed in the three mutants than in Col-0 plants at NaCl concentrations above 100mM or mannitol concentrations above 150mM (Fig. 2B, C).

This study also investigated whether the loss of function of CKA4 and CKA3 CKA4 affected the ABA-mediated root growth inhibition. High concentrations of ABA are known to inhibit root growth (Sharp and LeNoble, 2002). Germinated Col-0 and mutant seedlings were grown on MS agar plates containing different concentrations of ABA (0, 10, or 40 µM). The lengths of the primary roots of these plants were measured after 7 d of growth. As shown in Fig. 2D, the *cka4* and *cka3 cka4* mutants grew significantly longer roots than Col-0 seedlings on medium containing 10 µM ABA, indicating that the sensitivity of seedling root growth to ABA was reduced in the mutants.

### Disruption of CKA4 increases stomatal aperture and leaf water loss rate

**Fig. 3.**
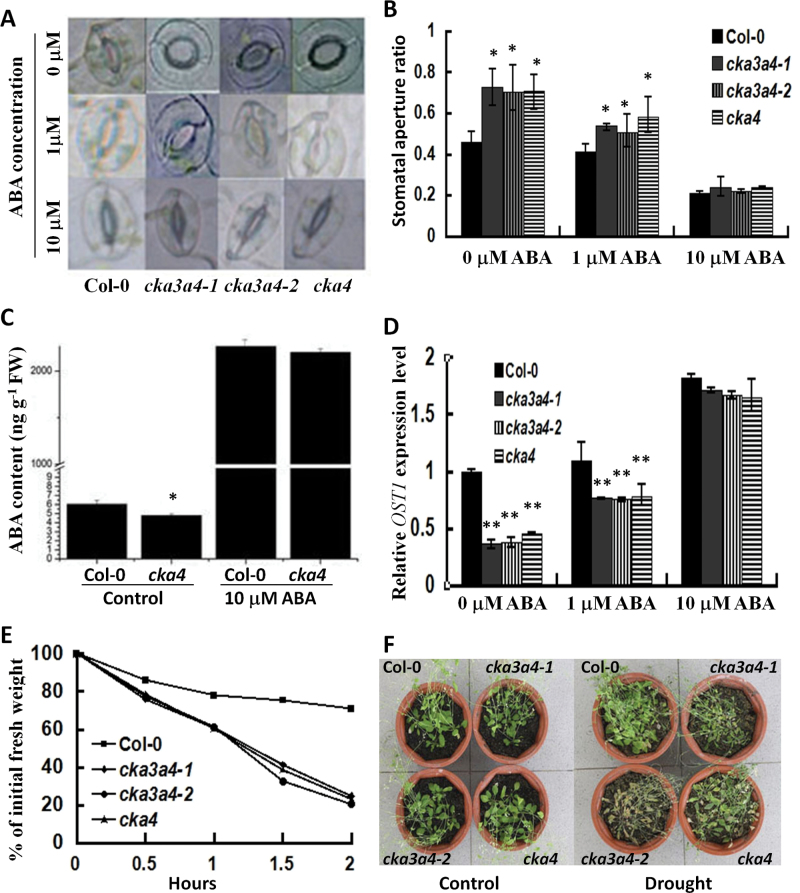
Changes in stomatal aperture, ABA content, *OST1* expression, and leaf water loss in *cka4* and *cka3 cka4* mutants. (A) Representative stomata in rosette leaves (from 30-d-old plants) with or without ABA treatment for 4h. (B) Stomatal aperture ratios (ratios of stomatal pore width to length) in rosette leaves with or without ABA treatment. Values of means±SD of three independent experiments with 40–50 stomata analysed in each experiment. (C) ABA levels in rosette leaves of 30-d-old plants with or without ABA treatment for 4h. Values are means±SD of three biological replicates. FW, fresh weight. (D) Relative *OST1* expression in rosette leaves of 30-d-old plants. Plants were treated with or without ABA for 4h. Values are means±SD of three biological replicates. (E) Water loss from detached rosette leaves of 30-d-old plants. Data are normalized to the initial leaf weight immediately after detachment. Values are means±SD of three biological replicates. (F) Plant status in a whole-plant water-loss experiment. Plants were well watered (control) or drought stressed by withholding water for 18 d. Asterisks indicate significant differences between mutant and Col-0 plants (*P<0.05, **P<0.01).

Each stoma is surrounded by two guard cells, which control the opening and closure of the stomatal aperture by their relative turgor pressure. ABA is known to play an important role in controlling stomatal aperture. To elucidate the potential role of CKA4 in stomatal regulation and the additive role of CKA3, the stomatal aperture of rosettes leaves was examined in *cka4* and *cka3 cka4* mutants. As shown in Fig. 3A and B, the *cka4* mutant had a significantly wider stomatal aperture size than Col-0 plants with 0 or 1 µM ABA treatment. However, the stomata of the mutant were still able to close effectively at the high ABA concentration (10 µM). Similar results were observed in the *cka3 cka4* double mutants.

The large stomatal aperture in the *cka4* and *cka3 cka4* mutants without ABA treatment might be attributed to either a deficiency in endogenous ABA or to ABA insensitivity. Therefore, the ABA levels were measured in the leaves of Col-0 plants and the *cka4* mutant with or without 10 μM ABA treatment. As shown in Fig. 3C, there was a slight but statistically significant difference in the leaf ABA levels between Col-0 and the *cka4* mutant without ABA treatment. However, no difference was observed between Col-0 and the *cka4* mutant with 10 μM ABA treatment for 4h.

Stomatal closure is known to be controlled by OPEN STOMATA 1 (OST1) and the expression level of *OST1* is regulated by ABA (Mustilli *et al.*, 2002). Quantitative RT-PCR analysis showed that the constitutive expression level of *OST1* in three mutants was markedly lower than that of Col-0 (Fig. 3D). The reduction in the *OST1* expression level in these mutants was still observed at a low ABA concentration (1 µM). At the high ABA concentration, no difference was observed between the mutants and wild-type plants. These expression results are in line with the increased stomatal aperture sizes in the mutants without or with a low ABA concentration treatment.

The stomatal aperture size was expected to be positively associated with the water-loss rate of plant leaves. To provide further supporting evidence for the effect of CKA4 mutation on stomatal aperture, leaf water loss was measured in the mutants and wild-type plants over a 2h period. The leaf water loss in the three mutants was markedly quicker than in Col-0 plants (Fig. 3E). Furthermore, water loss at the whole-plant level was also examined. In the well-watered conditions, no obvious difference was observed in growth between the mutants and Col-0 (Fig. 3F). With withdrawal of water supply, the plants of the three mutants in pots utilized water more quickly than Col-0 plants, which caused the mutant plants to wilt and wither earlier than Col-0 plants (Fig. 3F).

### CKA4 is upregulated by ABA, and CKA4 or CKA3 CKA4 mutation attenuates upregulation of ABA-responsive genes by ABA

**Fig. 4.**
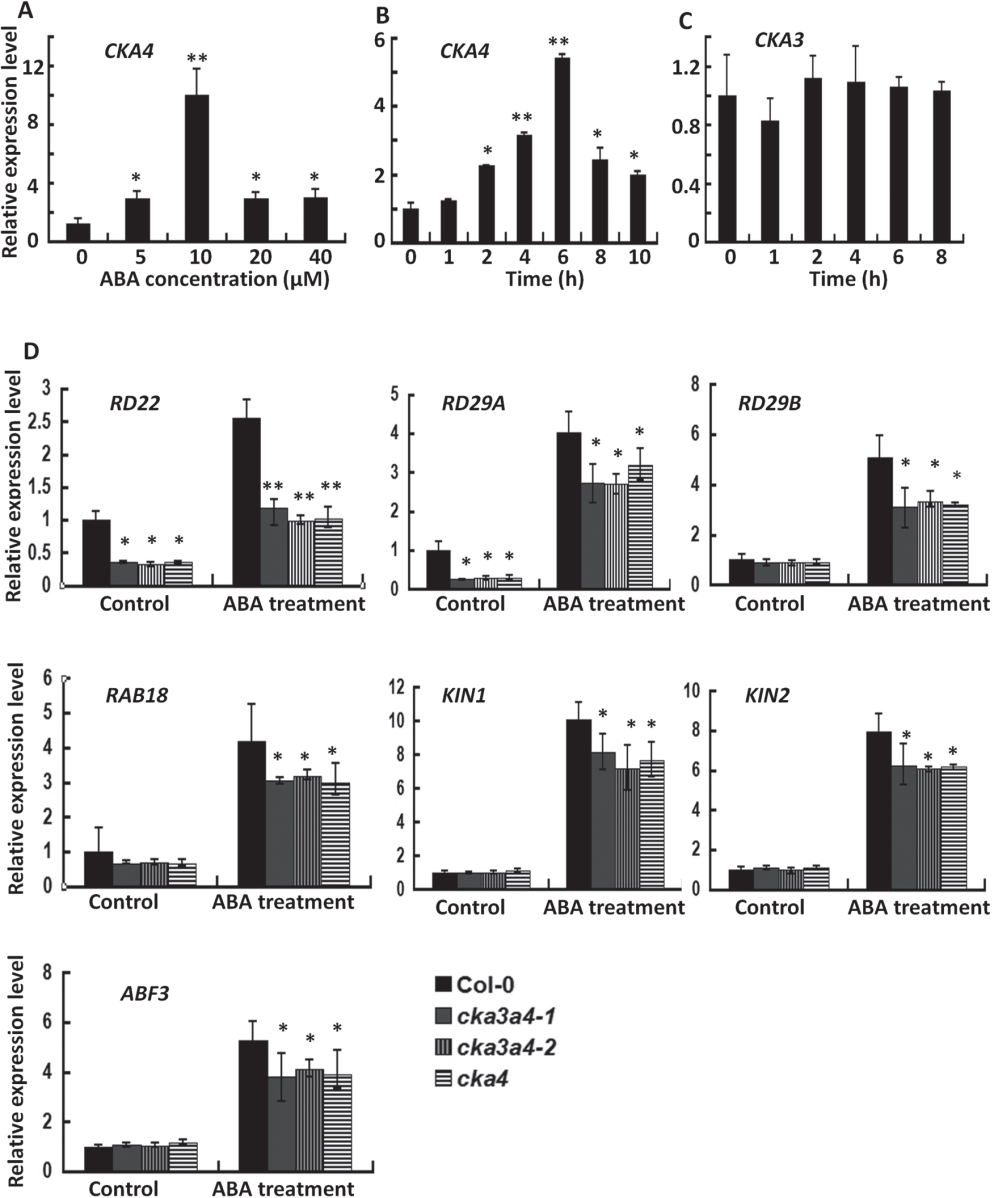
ABA-mediated expression changes of *CKA4*, *CKA3*, and potential downstream genes in the shoots of 2-week-old plants. (A) Relative *CKA4* expression level in Col-0 plants treated with various concentrations of ABA for 6h. (B) Relative *CKA4* expression level in Col-0 plants treated with 10 µM ABA for up to 10h. (C) Relative *CKA3* expression level in Col-0 plants treated with 10 µM ABA for up to 8h. (D) A comparative expression analysis of ABA-responsive genes in the seedlings of Col-0 and mutants treated with 10 µM ABA for 6h. Values are means±SD of three biological replicates. In (B) and (C), the relative expression level in ABA-treated samples was normalized to the sample without treatment (control) at each time point. In (D), the relative expression level was normalized to Col-0 under control conditions. Asterisks indicate significant differences between mutant and Col-0 or between ABA treatment and control (*P<0.05, **P<0.01).

To investigate whether CKA4 is involved in ABA-mediated gene regulation, the ABA responsiveness of *CKA4* and the effect of *CKA4* and *CKA3 CKA4* mutation on the expression of ABA-responsive genes were examined. *CKA4* expression was upregulated by ABA in Col-0 plants (Fig. 4A, B). A maximum upregulation of *CKA4* expression by ABA was seen at 10 µM and 6h after ABA treatment in 2-week-old Col-0 plants. In contrast, *CKA3* was not regulated by ABA in Col-0 (Fig. 4C). For the investigation of CKA4 involvement in regulation of genes in ABA signalling pathway, the expression levels of six ABA signalling downstream genes [*RESPONSIVE TO DEHYDRATION* (*RD*) *29A*, *RD29B*, *RD22*, *RESPONSE TO ABA 18* (*RAB18*), *KIN1*, and *KIN2*] were compared between the Col-0 and mutant plants after 10 µM ABA treatment for 6h. *RD29A*, *RD29B*, *RD22*, and *RAB18* are frequently used as ABA signalling downstream marker genes (Lång and Palva, 1992; Lee *et al.*, 2010). *Arabidopsis KIN1* and *KIN2* are cold- and ABA-inducible downstream genes and encode small polypeptides (Kurkela and Borg-Franck, 1992). Expression analysis revealed that induction of the six ABA-responsive marker genes by ABA was attenuated in the *cka4* and *cka3 cka4* mutants (Fig. 4D). The expression level of an *ABRE BINDING FACTOR3* (*ABF3*) was also lower in the mutant lines than in Col-0 (Fig. 4D), which is one of the important transcriptional activators of many ABA-upregulated downstream genes in vegetative organs and has been shown to upregulate *RD29* and *RAB18* (Kang *et al.*, 2002). These results indicated that CKA4 is a positive regulator in ABA signalling.

### cka3 and cka3 cka4 mutants show reduced thermotolerance

**Fig. 5.**
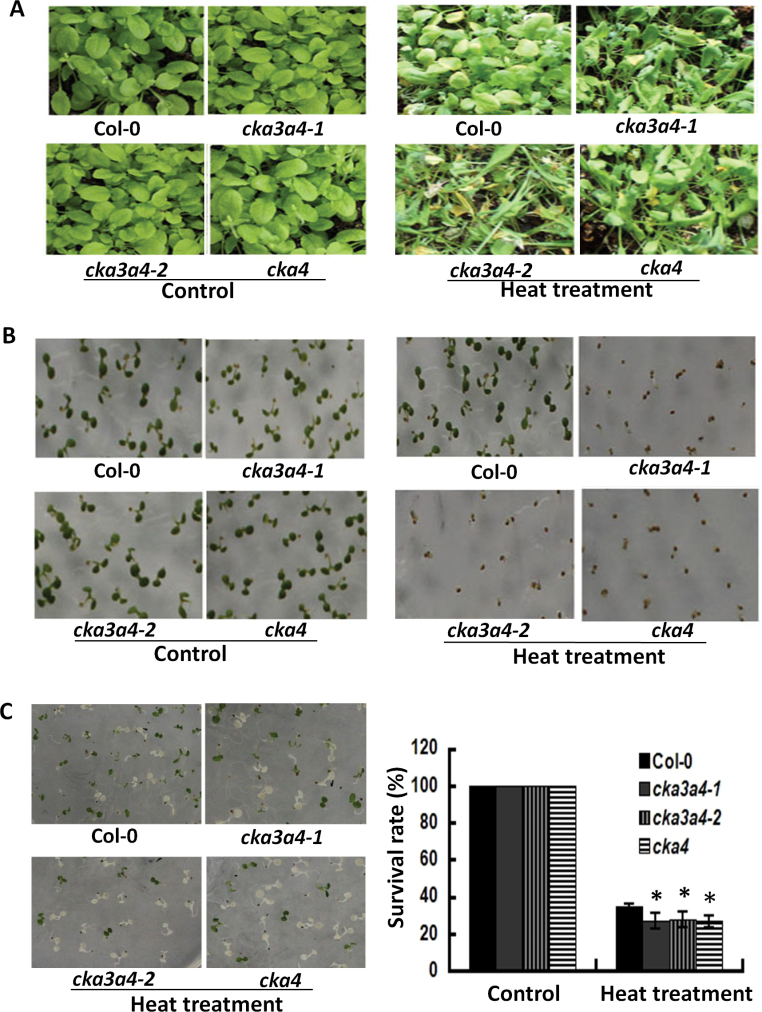
Reduced thermotolerance in *cka4* and *cka3 cka4* mutants. (A) Thermotolerance in 4-week-old plants. Four-week-old mutant and Col-0 plants were subjected to a heat-stress regime (37 °C for 2h, 22 °C for 2h, and 45 °C for 2.5h), followed by a 5 d recovery at 22 °C. No differences in the growth between Col-0 and *cka3 cka4* or *cka4* mutants at 22 °C (control) were observed. (B) Thermotolerance in young seedlings. Two-day-old germinated seeds were heated directly at 45 °C for 90min, followed by a 5 d recovery at 22 °C. (C) Survival rates of Col-0, *cka3 cka4*, and *cka4* mutants. Two-day-old seedlings were subjected to the heat-stress regime followed by a 5 d recovery at 22 °C. Values are means±SD of 300 plants (*P<0.05).

The involvement of CKA4 and the possible additive role of CKA3 in plant adaptation to heat stress were investigated in the *cka4* and *cka3 cka4* mutants. Three experiments were performed to evaluate the thermosensitivity of the mutants relative to Col-0 wild-type plants. In the first experiment, 4-week-old seedlings were exposed to a heat-treatment regime with heat acclimation (37 °C for 2h, 22 °C for 2h, and 45 °C for 2.5h). As shown in Fig. 5A, the *cka4* and *cka3 cka4* mutants were more sensitive to heat stress, showing severer damage by heat stress than Col-0. This indicates that the acquired thermotolerance is impaired in these mutants. In the second experiment 2-d-old germinated seeds were directly exposed to high temperature (45 °C for 90min), which is commonly used to assess basal thermotolerance. This treatment resulted in much severer growth retardation and mortality in the mutants compared with Col-0 (Fig. 5B), demonstrating the reduced basal thermotolerance in the mutants. The third experiment was to provide statistical data on the survival rates after heat stress between the mutants and Col-0 plants by exposing 2-d-old seedlings to the heat-treatment regime with heat acclimation, as described for the first experiment. The *cka4* and *cka3 cka4* mutants showed significantly lower survival rates than Col-0 (Fig. 5C). It appeared that the *cka3 cka4* double mutants had a similar survival rate to the *cka4* mutant, indicating that there was no additive effect of CKA3 to the reduced thermotolerance caused by the CKA4 loss-of-function mutation.

To investigate further the role of CKA4 in acquired thermotolerance, root and hypocotyl elongation assays were performed. No significant differences in root and hypocotyl elongation were observed between Col-0 plants and the three mutants under control conditions. Exposure of 5-d-old seedlings to the heat-treatment regime with heat acclimation as described above reduced root elongation in both Col-0 and the three mutants (Supplementary Fig. S2A, B at *JXB* online). The root-length reduction in the *cka4* and *cka3 cka4* mutants was more pronounced than that in Col-0 plants. The reduction of the hypocotyl length was also observed in the three mutants, but not in the wild-type seedlings in this heat-stress regime (Supplementary Fig. S2C, D). These results demonstrated that CKA4 or CKA3 CKA4 mutation causes significant impairment of root and hypocotyl elongation after heat stress.

### cka4 and cka3 cka4 mutants show reduced proline accumulation and increased MDA content after heat stress

**Fig. 6.**
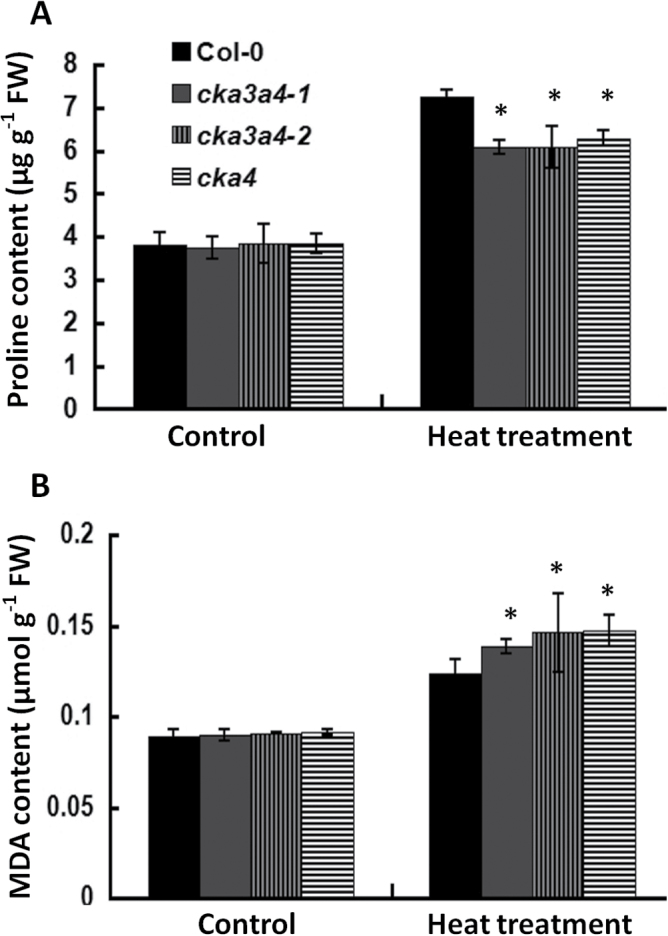
Accumulation of proline and MDA in Col-0 and *cka4* and *cka3 cka4* mutants after heat stress. (A) Proline content in the leaves of 30-d-old plants of Col-0 and mutants with or without a heat-treatment regime (37 °C for 2h, 22 °C for 2h, and 45 °C for 2.5h). (B) MDA content in the leaves of 30-d-old plants of Col-0 and mutants with or without the heat-treatment regime. Samples were harvested immediately after the heat-treatment regime. Values are means±SD of three biological replicates. Asterisks indicate significant differences between the mutants and the Col-0 (*P<0.05).

Increased proline accumulation is known to occur in plants under abiotic stress conditions, mainly for stress protection (Szabados and Savouré, 2010). High temperature can lead to increased lipid peroxidation, which is quantified by measuring the amount of MDA (Hulbert *et al.*, 2007). Therefore, proline and MDA contents were measured in Col-0 and three mutant plants after heat stress. No differences in the proline and MDA contents were observed in the leaves of 1-month-old plants between Col-0 and the mutants under non-stress conditions (Fig. 6). Both proline and MDA contents increased in Col-0 and the three mutants after heat stress. However, the *cka4* and *cka3 cka4* mutants accumulated significantly less proline in the leaves than Col-0 plants (Fig. 6A), which may indicate a reduced stress-protective capacity in the mutants. Significantly higher lipid peroxidation occurred in the mutants after heat stress, as indicated by the higher MDA content in the mutants than in Col-0 (Fig. 6B). These data demonstrated the relative severity of heat-stress damage in the cell membrane between the mutants and Col-0.

### CKA4 expression is upregulated during heat stress and recovery phase, and disruption of CKA4 reduces expression of heat-induced genes

**Fig. 7.**
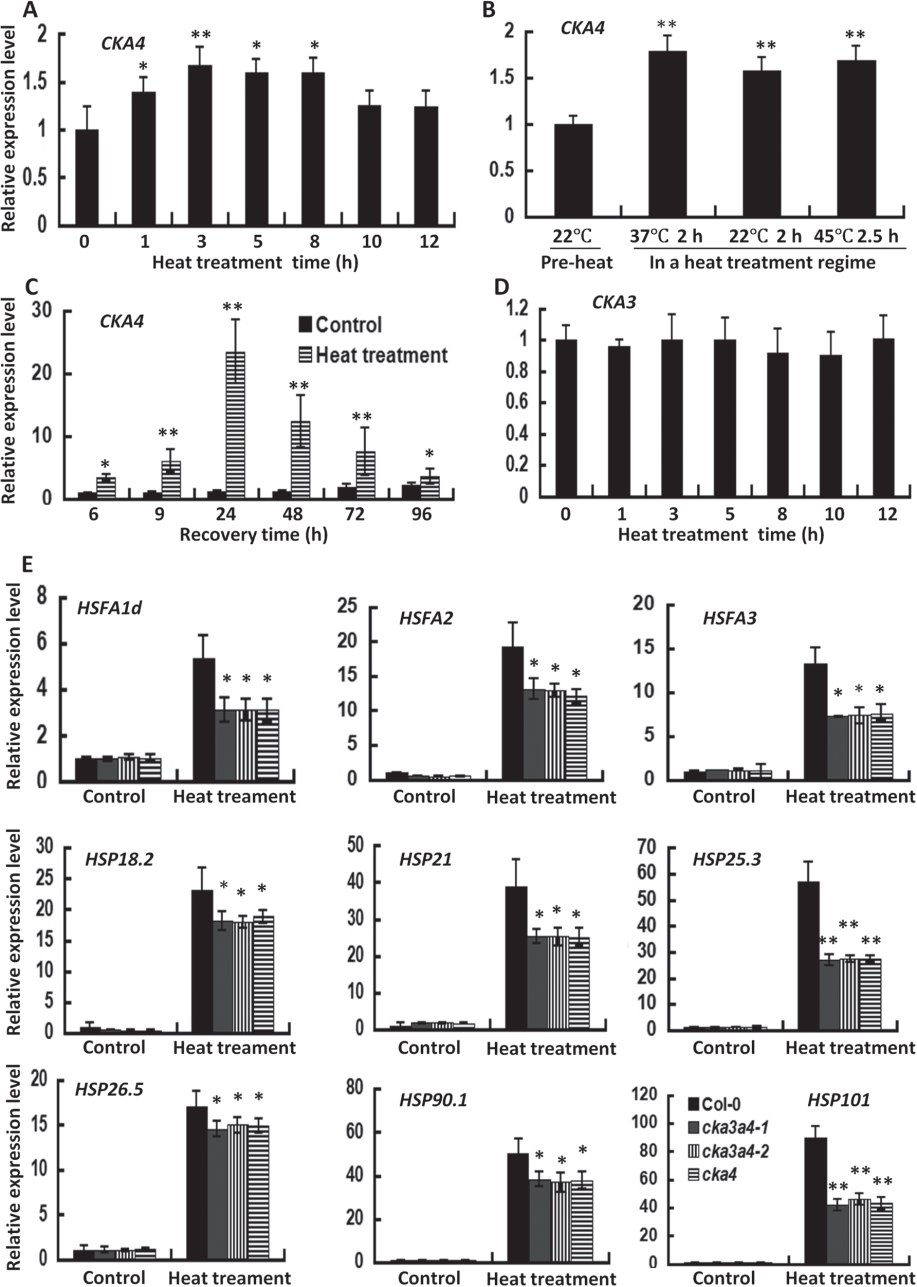
Expression levels of *CKA4*, *CKA3*, HSF, and HSP genes in the leaves of 4-week-old plants (A) Expression levels of *CKA4* in Col-0 plants during heat treatment (37 °C). (B) Expression levels of *CKA4* in Col-0 plants during a heat-treatment regime (37 °C for 2h, 22 °C for 2h and 45 °C for 2.5h). Pre-heat samples were harvested before heat treatment. (C) Expression levels of *CKA4* in Col-0 plants during the recovery phase. Samples were harvested for analysis during the 22 °C recovery phase after the heat-treatment regime. (D) Expression levels of *CKA3* in Col-0 plants during heat treatment (37 °C). (E) Expression levels of HSF and HSP genes in Col-0 plants and mutants treated at 37 °C for 3h. Control samples were without heat treatment. In (A)–(D), relative expression levels in treated samples was normalized to samples without treatment (control) at each time point. In (E), the relative expression level was normalized to Col-0 plants under control conditions. Values are means±SD of three biological replicates. Asterisks indicate significant differences between treated and control or between mutant and Col-0 plants (*P<0.05, **P<0.01).

To gain insights into the molecular mechanisms of reduced thermotolerance caused by *CKA4* mutation, the expression response of *CKA4* to heat stress and a comparative expression analysis of representative HSF and HSP genes were performed. Quantitative RT-PCR analysis showed that *CKA4* expression in the leaves of Col-0 plants was significantly upregulated during heat acclimation (Fig. 7A, B). The *CKA4* mRNA level remained elevated after a 2h recovery at 22 °C in the heat-treatment regime adopted in this study (Fig. 7B). Most interestingly, a very high level of *CKA4* transcript accumulation was observed during the recovery phase, 24h after the heat-treatment regime (Fig. 7C). No significant change in the expression of *CKA3* during heat stress was observed in Col-0 plants (Fig. 7D).

During heat acclimation, the expression levels of HSFs (*HSFA1d*, *HSFA2*, and *HSFA3*) and HSPs (*HSP18.2*, *HSP21*, *HSP25.3*, *HSP26.5*, *HSP90.1*, and *HSP101*) were markedly upregulated (Fig. 7E). This heat-induced upregulation was apparently less in *cka4* and *cka3 cka4* mutants than in Col-0 (Fig. 7E). The attenuation in the heat-induced upregulation of *HSP25.3* and *HSP101* transcripts in the mutants was particularly pronounced. A decrease in the accumulation of these gene transcripts in the mutants indicated a reduced thermotolerance capacity in these plants. Again, no significant differences in the heat-induced transcript accumulation of these HSF and HSP genes were observed between *cka4* and *cka3 cka4* double mutants, suggesting no additive role of CKA3 to CKA4 in the heat-mediated regulation of these heat-stress genes.

### CKA4 knockout reduces the expression of critical genes involved in retrograde signalling pathways and some plastid-encoded RNA polymerase target genes

**Fig. 8.**
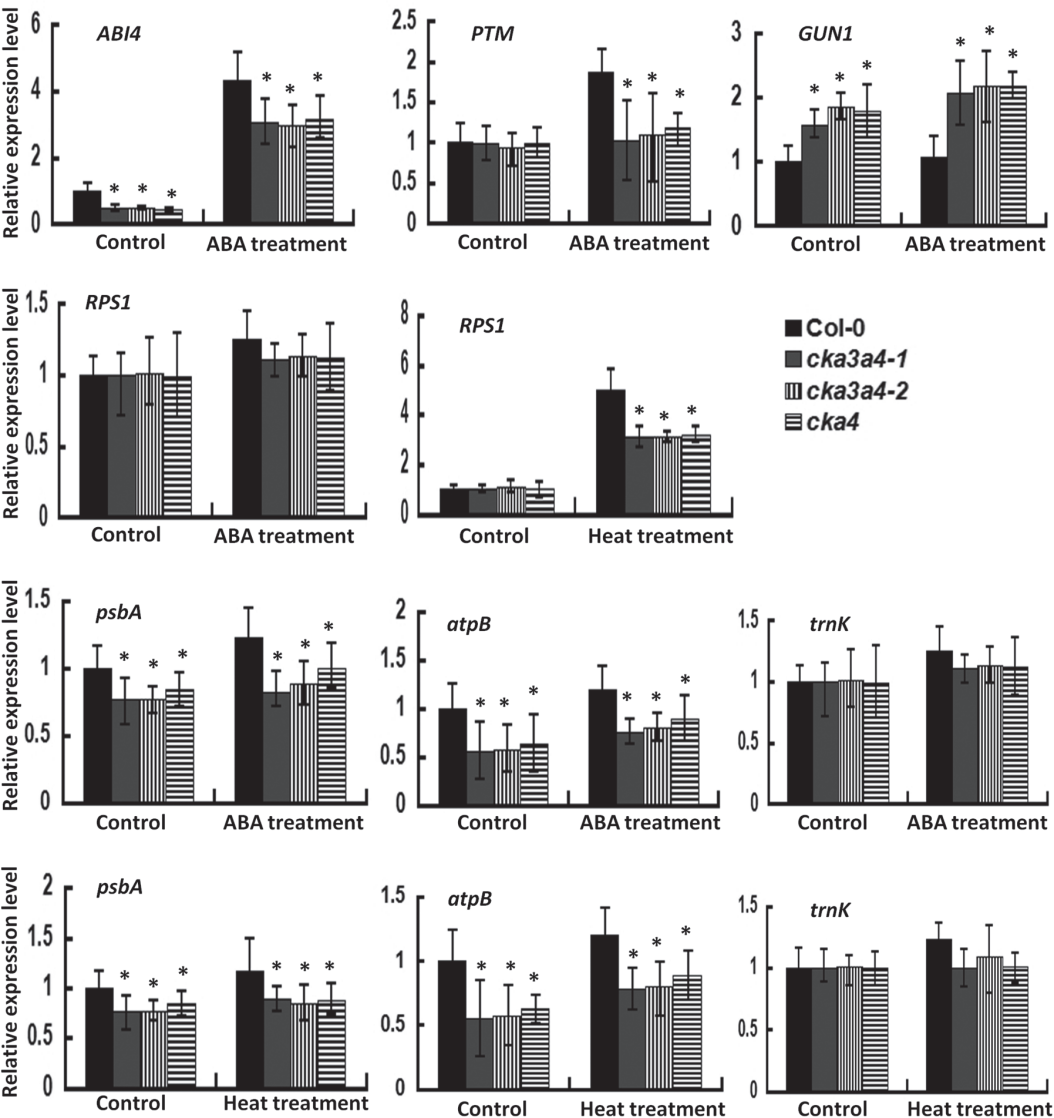
Expression levels of genes involved in retrograde signalling pathways and plastid-encoded RNA polymerase target genes in Col-0, *cka4*, and *cka3 cka4* mutants. The leaves of 4-week-old plants were used for analysis. Plants were treated with either 10 µM ABA for 6h or at 37 °C for 3h in comparison with no treatment (control). Relative expression level was normalized to Col-0 under control conditions and values are means±SD of three biological replicates. Asterisks indicate significant differences between mutant and Col-0 plants (*P<0.05).

Alterations in the expression levels of nuclear genes in the *cka4* mutant suggested that CKA4 is involved in retrograde signalling from the chloroplast to the nucleus. One known retrograde signalling pathway involves GEMONES UNCOUPLED 1 (GUN1, a plastid RNA-binding protein), a PHD-type transcription factor with a transmembrane domain (PTM) and ABI4; ABI4 is a central regulator for this retrograde signalling and is also involved in the ABA signalling pathway (León *et al.*, 2013). To investigate whether genes involved in this retrograde signalling pathway are affected by the *CKA4* mutation, the expression levels of these three genes in the mutant and Col-0 plants under normal and ABA-treated conditions were determined. As shown in Fig. 8, the expression level of *ABI4* was significantly reduced in the *cka4* and *cka3 cka4* mutants under both normal conditions and ABA treatment. A significant reduction in *PTM* expression was also observed in the mutants with ABA treatment but not in the normal conditions. In contrast, the expression level of *GUN1* was higher in the mutants than in Col-0 in both control and ABA-treated plants.

For retrograde signalling during heat stress, a heat-responsive nucleus-encoded plastid ribosomal protein S1 (PRS1) has been shown to be involved in this process (Yu *et al.*, 2012). This study also showed that the expression level of *PRS1* was elevated in heat-treated plants (Fig. 8), but its heat-induced upregulation was attenuated in the *cka4* and *cka3 cka4* mutants. No significant differences in the expression levels of *PRS1* under control conditions and ABA treatment were observed between Col-0 and the mutants.

Plastid CK2 is known to phosphorylate some components of plastid-encoded RNA polymerase (PEP) complex and has been proposed to have a role in regulating expression of some plastid-encoded genes (Schweer *et al.*, 2010*b*). The availability of the *cka4* mutant provided us an opportunity to test this hypothesis. This study analysed the expression levels of three known PEP target genes [*psbA* coding for photosystem II D1 protein, *atpB* coding for the β-subunit of chloroplast ATPase complex, and *trnK* (tRNA-Lys); Schweer *et al.*, 2010*b*]. Expression levels of *psbA* and *atpB* were significantly reduced in the *cka4* and *cka3 cka4* mutants under normal conditions as well as with ABA and heat treatment in comparison with Col-0 plants, while *trnK* expression was not affected (Fig. 8).

### cka4 and cka3 cka4 mutants show normal growth phenotypes under non-stress conditions

In order to see whether the reduced ABA sensitivity and thermotolerance was due to alteration in growth as a result of *CKA4* mutation, a comparative growth analysis was performed. As shown in Supplementary Fig. S3 at *JXB* online, no growth defects were observed in the *cka4* and *cka3 cka4* mutants. The morphologic appearance, rosette size and shoot and root dry matter production in 4-week-old plants were similar between Col-0 and three mutants. These data indicated that *CKA4* knockout mutation does not affect plant morphology and growth under normal growth conditions.

## Discussion

This study showed that a knockout mutation in the plastid CK2 gene (*CKA4*) resulted in reduced ABA sensitivity and thermotolerance in *Arabidopsis*, which was further supported by the same phenotypes observed in two *cka3 cka4* double-knockout mutants. Alterations in these phenotypes in the *cka4* and *cka3 cka4* mutants were accompanied by a reduction in signal transduction for inducing expression of ABA- or heat-stress-responsive downstream genes in the nucleus. This indicates that the phosphorylation process by plastid CK2 enhances retrograde signalling from the plastid to the nucleus. Thus, this study demonstrated that plastid CK2 is a positive regulator in retrograde signalling in the plant response to ABA and heat stress.

A previous knockout mutation study has shown that three nuclear-located CK2 α-subunits (CKA1–3) in *Arabidopsis* have a synergistic role in ABA-induced blockage of seed germination and cotyledon greening (Mulekar *et al.*, 2012), but the molecular basis of this ABA insensitivity in these mutants was not investigated. Although the upstream pathway of nuclear CK2 involvement leading to this ABA insensitivity is expected to differ from the plastid CK2, we thought that *cka3 cka4* double mutants might have severer ABA-insensitivity phenotypes during seed germination and seedling growth. However, no significant differences in the phenotypes and expression levels of downstream genes were observed between the *cka4* mutant and *cka3 cka4* mutants in the plant response to ABA. Similarly, the reduced thermotolerance and expression of heat-responsive genes in the *cka4* mutant did not differ significantly from those in the *cka3 cka4* double mutants during heat stress. This indicates that plastid CK2, through retrograde signalling, may play a dominant role over the nuclear CKA3 in regulating gene expression during the processes investigated. However, whether nuclear CK2 α-subunits in plants are involved in signal transduction during heat stress is currently unknown, although mammalian nuclear CK2 phosphorylates HSF1 (Soncin *et al.*, 2003), a key regulator in the mammalian cell heat response. *CKA4* has been shown to have the highest expression level among four α-subunit genes, with *CKA3* being the lowest in the leaf, root, stem, and flower organs of *Arabidopsis* (Salinas *et al.*, 2006). In particular, the function of the three nuclear CK2 α-subunit genes is likely to be redundant. This could be one factor for contributing to the lack of observable additive effect of CKA3 seen in the *cka3 cka4* double mutant lines. However, the two *cka3 cka4* double mutants used in this study can serve as two independent lines for confirming the phenotypic changes resulting from *CKA4* mutation, as these double mutants have a *CKA4* knockout mutation by T-DNA insertion at two different locations from the *cka4* mutant.

ABA regulates many important plant processes such as seed germination, dormancy, seedling growth, and stomatal closure (Finkelstein *et al.*, 2002; Mustilli *et al.*, 2002; Fujii *et al.*, 2007). The molecular mechanisms underlying ABA responses in plants have been intensively studied by biochemical and genetic approaches (Pandey *et al.*, 2005). The major molecular players in the core ABA signalling network have been identified by molecular and genetic analyses (Fujii *et al.*, 2009; Umezawa *et al.*, 2010; Raghavendra *et al.*, 2010; Nakashima and Yamaguchi-Shinozaki, 2013). The reduced sensitivity phenotypes of the *cka4* mutant in response to exogenous ABA during seed germination and seedling growth indicate that CKA4 is involved in modulating ABA signalling. *CKA4* expression was upregulated by ABA, which may serve as enhancement for its action in this process. The reduced ABA sensitivity in the *cka4* mutant is particularly evident in the following two datasets: (i) significantly reduced inhibition of root growth in the mutants in the presence of 10 μM ABA (Fig. 2D), and (ii) a significant reduction in the ABA-mediated upregulation of six nuclear-encoded ABA-responsive downstream genes (*RD22*, *RD29A*, *RD29B*, *RAB18*, *KIN1*, and *KIN2*) in an experiment where 10 μM ABA was used for induction (Fig. 4D). A significant reduction in the expression level of *ABF3* was also observed in the *cka4* mutant. ABF3 is an important transcriptional activator in vegetative organs and binds to ABA-responsive elements (Yoshida *et al.*, 2010), which are present in many downstream genes (e.g. *RD29B* and *RAB18*) of the ABA regulatory network (Kang *et al.*, 2002).

Interestingly, the stomatal apertures of the *cka4* mutant were significantly wider than those of Col-0 plants under normal conditions and following treatment with a low concentration of ABA. Expression analysis revealed that the expression level of *OST1* was significantly lower in the *cka4* mutant than in Col-0 under these conditions. OST1, expressed in stomatal guard cells and vascular tissue, positively regulates ABA- and reactive oxygen species-mediated stomatal closure (Mustilli *et al.*, 2002). OST1 controls the activity of guard cell anion channel SLAC1 (Geiger *et al.*, 2009). Because of the wider opening of the stomata in the mutant, the leaf water loss in the mutant was more rapid than in Col-0 and the mutant became more susceptible to drought stress. The wider opening of the stomatal aperture in the *cka4* mutant than in Col-0 under normal conditions appears to be related at least partly to its lower leaf ABA level. As part of the ABA synthetic pathways is located in the chloroplast (Seo and Koshiba, 2002), the *CKA4* knockout may also affect the activities of some enzymes involved in the chloroplast part of the ABA synthetic pathways. However, the *CKA4* knockout did not affect plant growth under normal growth conditions.

In addition to its role in ABA signalling, this study showed that CKA4 also participated in heat-stress signalling. Knockout mutation of *CKA4* resulted in increased plant susceptibility to heat stress and reduced the survival rate of plants after exposure to a lethally high temperature. Both basal and acquired thermotolerance capacities were affected by *CKA4* mutation. The root and hypocotyl elongation rates after heat treatment were significantly reduced in the *cka4* mutant, indicating that more severe heat injury occurred in the mutant than in Col-0. The *cka4* mutant plants accumulated more MDA after heat stress. MDA is a secondary end product of the oxidation of polyunsaturated fatty acids and is considered an indicator of general lipid peroxidation (Hodges *et al.*, 1999). Thus, the increased MDA level indicates increased lipid peroxidation in the mutant line after heat treatment. The *cka4* mutant plants accumulated less proline during heat stress than Col-0. Proline has long been recognized as one of the general abiotic stress protectants. It functions as a molecular chaperone, is able to protect protein integrity, and enhances the activities of many enzymes (Szabados and Savouré, 2010), which can be beneficial for plant recovery from heat stress. The reduced level of proline in the *cka4* mutant may indicate that the general protection system is impaired due to the absence of the plastid CK2.

The reduced thermotolerance in the *cka4* mutant may be partly attributed to impairment in ABA signalling, as it is known that some ABA-deficient (*aba1*, *aba2*, and *aba3*) and -insensitive (*abi1*, *abi2*, and *abi3*) mutants are more sensitive to heat stress (Larkindale *et al.*, 2005; Penfield, 2008). However, the HSP levels in these ABA-deficient and -insensitive mutants are unaffected (Larkindale *et al.*, 2005). In contrast, *cka4* mutation resulted in reduced expression levels of HSPs, indicating that the impact of CKA4 on thermotolerance also involves a unique pathway. It is known that HSPs have an important protective role during heat stress (Wang *et al.*, 2004; Zhang *et al.*, 2009). HSPs accumulate during the heat-acclimation stage for plants to acquire some degree of thermotolerance to lethally high temperatures (Wang *et al.*, 2004). Increased levels of HSP accumulation in plants can enhance thermotolerance (Bita and Gerats, 2013; Grover *et al.*, 2013). HSFs are central regulators of HSP genes (Pirkkala *et al.*, 2001; Scharf *et al.*, 2012; Xue *et al.*, 2014). In this study, the heat-induced expression levels of six representative HSP genes were found to be lower in the *cka4* mutant than in Col-0, which explains the low thermotolerance in the mutant. The low expression levels of HSP genes were accompanied by reduced expression levels of three HSFs analysed (*HSFA1d*, *HSFA2*, and *HSFA3*), suggesting that CKA4 is a positive regulator of the heat-acclimation process, likely to be upstream of the HSF genes.

The *CKA4* transcript was also upregulated during heat stress. Interestingly, the *CKA4* transcript level was markedly elevated during the recovery phase and peaked at 24h after the completion of heat treatment. Deregulation of some genes days after heat exposure has been observed in the previous studies (Pecinka and Schield, 2012; Popova *et al.*, 2013). This study also showed that the transcript level of *HSP101* remained elevated 24h after heat treatment and that the post-heat transcript level of *HSP101* was lower in the *cka4* and *cka3 cka4* double mutants than in Col-0 (Supplementary Fig. S4 at *JXB* online). HSP101 is considered to be one of the HSPs playing a key role in the acquisition of thermotolerance in plants (Gurley, 2000; Queitsch *et al.*, 2000). It is possible that the higher levels of HSPs during the post-heat-stress period can also help plant recovery from heat injury.

Another possible role of CKA4 in protecting plants from heat injury is that CKA4 may be involved in phosphorylation of HSPs in the chloroplast. Phosphorylation of some HSPs by CK2 has been observed in the mammalian system (Lees-Miller and Anderson, 1989; Ishihara *et al.*, 2000). In plants, a number of HSPs are present in the chloroplast (Chen and Vierling 1991; Shi and Theg, 2010) and are known to play important roles in protecting photosynthetic apparatus (Chauhan *et al.*, 2012). Examination of the high-confidence list of identified chloroplast phosphoproteins reveals that no chloroplast HSPs are phosphorylated (Reiland *et al.*, 2009), indicating that this is an unlikely route for the observed impact of CKA4 on thermotolerance.

Our findings on the loss-of-function mutation of a plastid CK2 resulting in a reduction in ABA- or heat-induced expression of many nuclear genes suggest that CKA4 has a role in retrograde signalling. It is known that the expression of many chloroplast proteins involved in photosynthesis is coordinately regulated in plants (Chi *et al.*, 2013). Such coordination is essential to achieve the balance of photosynthetic components, such as ribulose-1,5-bisphosphate carboxylase/oxygenase, which contains a plastid-encoded large subunit and a nuclear-encoded small subunit. Plastid CK2 has been shown to phosphorylate the components (e.g. SIG1 and SIG6) of the PEP complex (Ogrzewalla *et al.*, 2002; Schweer *et al.*, 2010*b*; Türkeri *et al.*, 2012), implicating its role in regulation of plastid gene expression. The phosphorylated *Arabidopsis* SIG6 (AtSIG6) has a higher binding affinity to the promoter of *Arabidopsis atpB* than the unphosphorylated protein (Türkeri *et al.*, 2012). Plastid CK2 phosphorylation of AtSIG6 occurs at multiple sites (Schweer *et al.*, 2010*a*). A complementation study of an *AtSIG6* knockout mutant with *AtSIG6* phosphorylation site mutants has shown that a mutation in many critical phosphorylation sites reduces the expression level of *atpB* in *Arabidopsis* but has no obvious effect on the expression of *psbA* based on non-quantitative northern blot hybridization analysis (Schweer *et al.*, 2010*a*). This study provides direct experimental evidence that the expression levels of two PEP target genes (*atpB* and *psbA*) were reduced by the knockout mutation of a plastid CK2. Although no obvious effect of *AtSIG6* phosphorylation site mutation on *psbA* expression was observed in the study of Schweer *et al.* (2010*a*), this might be due to the use of single-phosphorylation-site mutants in their study. The effect of mutations in multiple phosphorylation sites of ATSIG6 on *psbA* expression has not been tested to date. The alterations in the levels of some critical plastid proteins are likely to have some effect on the expression of nuclear genes. For example, the heat-stress-mediated upregulation of *HSFA2* and some HSP genes is inhibited by downregulation of a plastid ribosomal protein S1 (PRS1) (Yu *et al.*, 2012), which is involved in plastid protein synthesis. In fact *CKA4* knockout also reduced the expression of *PRS1*, indicating that CKA4 is likely to be an upstream regulator of *PRS1*, which subsequently influences the expression levels of some HSF and HSP genes. However, the pathway of this heat-stress retrograde signalling is currently unknown.

A well-known retrograde signalling pathway from the chloroplast to the nucleus involves GUN1, PTM, and ABI4. PTM and ABI4 are transcription factors. PTM is associated with the chloroplast envelope membrane and can move to the nucleus to activate the expression of *ABI4* (Chi *et al.*, 2013). In this retrograde signalling, ABI4 is a regulator for modulating expression of nuclear genes and is also involved in ABA and sugar signalling pathways (León *et al.*, 2013). The expression levels of *PTM* and *ABI4* were reduced in the *cka4* mutant with ABA treatment, suggesting that this retrograde signalling is modulated by CKA4. STN7 kinase is involved in the GUN1–PTM–ABI4 retrograde signalling pathway (Chi *et al.*, 2013) and is regulated by the redox state of chloroplast (Pesaresi *et al.*, 2009). STN7 is suggested to be a potential substrate of plastid CK2 (Reiland *et al.*, 2009). Whether CKA4 modulating the GUN1–PTM–ABI4 retrograde signalling pathway is through phosphorylation of STN7 awaits further investigation. Furthermore, plastid CK2 is also regulated by the redox state chloroplast (Pfannschmidt and Liere, 2005). Thus, CKA4 may have a role in linking the redox signal in the chloroplast to ABA and heat-stress signalling in the nucleus.

In summary, the present study provides a substantial body of work that supports a role for the plastid CK2, CKA4, in positive regulation in the ABA and heat-stress signalling pathways. Knockout mutation of *CKA4* led to reduced sensitivity in the ABA-mediated blockage of seed germination and seedling growth and reduced thermotolerance. These phenotypes observed in the *cka4* mutants were attributed to its function in modulating the expression levels of ABA- and heat-responsive genes in the nucleus via retrograde signalling. *CKA4* mutation negatively influences retrograde signalling via the GUN1–PTM–ABI4 and PRS1 pathways. These findings provide an exciting opportunity for identification of the additional molecular players in ABA and heat-stress signalling pathways and exploitation of CKA4 as a potential modulator for improvement of plant tolerance to various abiotic stresses in the future.

## Supplementary data

Supplementary data are available at *JXB* online.

Supplemental Table S1. Sequences of oligonucleotide primers used in this study.

Supplementary Fig. S1. The expression level of *At1g66110* gene in Col-0 and *cka4* mutant (the progeny of CS311135).

Supplementary Fig. S2. Root and hypocotyl elongation in Col-0, *cka4*, and *cka3 cka4* mutants after heat treatment.

Supplementary Fig. S3. Comparative analysis of growth parameters of wild-type Col-0, *cka4*, and *cka3 cka4* mutant plants.

Supplementary Fig. S4. The expression level of *HSP101* at the 24h recovery phase after a heat-treatment regime.

Supplementary Data

## Abbreviations:

ABA: abscisic acid
CK2: casein kinase 2
HSF: heat-shock factor
HSP: heat-shock protein
LC-MS/MS: liquid chromatography-tandem mass spectrometry
MDA: malondialdehyde
MS: Murashige and Skoog
PTM: PHD-type transcription factor with a transmembrane domain
PEP: plastid-encoded RNA polymerase
RT-PCR: reverse transcription-PCR
SD: standard deviation.
