# Corrigendum for: Plastid casein kinase 2 knockout reduces abscisic acid (ABA) sensitivity, thermotolerance, and expression of ABA- and heat-stress-responsive nuclear genes

**DOI:** 10.1093/jxb/erv406

**Published:** 2015-08-18

Yu Wang^1,*^ Hongping Chang^1,*^ Shuai Hu^1^, Xiutao Lu^1^, Congying Yuan^1^, Chen Zhang^1^, Ping Wang^1^, Wenjun Xiao^1^, Langtao Xiao^2^, Gang-Ping Xue^3,†^ and Xinhong Guo^1,†^

^**1**^ State Key Laboratory of Chemo/Biosensing and Chemometrics, College of Biology, Hunan University, Changsha 410082, PR China

^**2**^ Hunan Provincial Key Laboratory of Phytohormones and Growth Development, Hunan Agricultural University, Changsha, 410128, PR China

^**3**^ CSIRO Plant Industry, 306 Carmody Road, St Lucia, QLD 4067, Australia

* These authors contributed equally to this work.

† To whom correspondence should be addressed. E-mail: Gang-Ping.Xue@csiro.au or gxh@hnu.edu.cn

*Journal of Experimental Botany*, Vol. 65, No. 15, pp. 4159–4175, 2014, doi:10.1093/jxb/eru190

During the assembly of Figure 5 the following mistakes were inadvertently made:

1. Figure 5A: An image representing the *cka3a4-2* mutant was mistakenly reused to also represent the *cka3a4-1* mutant. In addition, a subsection of the Col-0 image was mistakenly used to depict the *cka4* mutant.
2. Figure 5B: A subsection of the Col-0 control image was mistakenly used to depict the Col-0 heat treatment.
3. Figure 5C: A subsection of the heat treated Col-0 image was mistakenly used to represent the heat treated *cka3a4-1* mutant.

The corrected version of this figure can be found below:



