# Supplementary material for: Plastid casein kinase 2 knockout reduces abscisic acid (ABA) sensitivity, thermotolerance, and expression of ABA- and heat-stress-responsive nuclear genes
Source: J Exp Bot. 2014 May 6;65(15):4159–75. doi: 10.1093/jxb/eru190 (PMC4112627; doi:10.1093/jxb/eru190)
Supplement: Supplementary Data [file supp_65_15_4159__index.html]

Plastid casein kinase 2 knockout reduces abscisic acid (ABA) sensitivity, thermotolerance, and expression of ABA- and heat-stress-responsive nuclear genes — Plastid casein kinase 2 knockout reduces abscisic acid (ABA) sensitivity, thermotolerance, and expression of ABA- and heat-stress-responsive nuclear genes — Supplementary Data 

# Plastid casein kinase 2 knockout reduces abscisic acid (ABA) sensitivity, thermotolerance, and expression of ABA- and heat-stress-responsive nuclear genes

## Supplementary Data

Data files

**Files in this Data Supplement:**

- Supplementary Data - Supplementary Data
