# Supplementary material for: Plastid casein kinase 2 knockout reduces abscisic acid (ABA) sensitivity, thermotolerance, and expression of ABA- and heat-stress-responsive nuclear genes
Source: J Exp Bot. 2014 May 6;65(15):4159–75. doi: 10.1093/jxb/eru190 (PMC4112627; doi:10.1093/jxb/eru190)
Supplement: Supplementary Data [file supp_eru190_jexbot118893_file001.pdf]

Supplemental Data

Plastid protein kinase CK2 knockout reduces ABA sensitivity, thermotolerance and expression of ABA- and heat stress-responsive nuclear genes

Yu Wang, Hongping Chang, Shuai Hu, Xiutao Lu, Congying Yuan, Chen Zhang, Ping Wang, Wenjun Xiao, Langtao Xiao, Gang-Ping Xue, Xinhong Guo

Supplemental Table S1. Sequences of oligonucleotide primers used in this study

Gene name	F: Forward primer sequence(5′–3′)	R：Reverse primer sequence(5′–3′)
	**T-DNA screening primers**	
cka3-cka4-1	ACGTTGACATCAGCGTAAACC	GATCTTGATAACGCAACGCTC
cka3-cka4-2	AGAGTTAAAAAAAGACATTAATGGC	ACCAATCCCAAACCAATCTATA
cka4	TGCACAGAGATGTGAAACCAC	ATCATAGTCCTGTAAATCCACCA
	**Real time-PCR Primers**	
Actin-2	CACTGTGCCAATCTACGAGGGT	CACAAACGAGGGCTGGAACAAG
CKA3	TAGAGATGAGCACTCCAAAACA	AGTGACAGAAATCCAATGCC
CKA4	TCTTTTATGGCCATGACAACT	CTGTTGGCCTTTCTTGGT
At1G66110	TGCGTAAAGGGTTTGTTAGAGT	AACTTCACCAAGTCCATTAATC
RD22	TTCGGAAAAGCGGAGAT	CTTTGAAGGCCAAGTGGT
RD29A	AGGAACCACCACTCAACA	GCTCGTCATCATCATCATCT
RD29B	AAGGAGACGCAACAAGGG	ACGGTGGTGCCAAGTGAT
OST1	GGATCAACCGGGCCAAAG	TGAGTGCCTGCAGGAGGAA
KIN1	GTCAGAGACCAACAAGAATG	CGCATCCGATACACTCTT
KIN2	GGCCGCTGGCAAAGCT	GCAGCATCCTTGGCCTTGT
RAB18	AGCAGCAGTATGACGAGTA	CCACCTGTTCCGTATCCT
ABF3	AATGGCGGATTCTATGGATT	GCATCTGTAGTGGCTGAG
HSP101	ATTCACACACAAGACAAACGAG	ACCAAATCTCTTCCATAAGTCTT
HSP90.1	TTGCGTTGAATCAAAGTTCGTTGC	TGTCCTATCATGCTTACATCAGCTCC
HSP26.5	ATGGCTCTAGCTCGTCTGG	GTTGTAGTAACCATAACTCTTTGAA
HSP25.3	ATGGCTTCTACACTCTCATTTGCTGCATCGGCTC	AGAGACGTCCATGGTTAAGCGTTGTTGAGGTC
HSP21	GGCTCTATGTTCACCTCTTGC	CATAGTATCTAACATTTGTCGCATC
HSP18.2	GTCTTTGATCCGTTCTCGC	CGGTAACCTAAACCTTCTCATA
HSFA1d	TGACTTGGAATCAAAAATCCGAATGGATGTGAG	ACTGTTGTTATTAGCACTCCAAGAGACAATCG
HSFA2	GAAGGGCTTAACGAAACAGGGCCACCAC	GATCCTTGCTGATTCACATTCTGCAAACC
HSFA3	CTTGGGGACTGACCGGAGCTAGCTTCGTAG	GCTAGTGCTACTGCAGCAAGTTTGGTTG
ABI4	ATGGACCCTTTAGCTTCC	AACGCCACGGTAACGGAACT
PTM	TCTATTTTCCAAAGTGCTCCA	TTTCTCCATCTTGTCCCTCCT
GUN1	GCTTTTATGAGTTTGCGGTTA	ACAATCTTTCGTCGTTCGGTT
PSBA	GCCTATGGGGTCGCTTCTGTA	AGGAATAATGGCACCGGAAAT
RPS1	AACTGTTCTTCAGCCTGGTGACAC	CTGTCTGAATGTCTGAGCCATCTC
ATPB	AATGCTCTGGTGGTTAAGGGT	CTGTCGCACTCATAGCTACAG
TRNK	TAACTCAACGGTAGAGTACTCGGCT	ACTCGAACCCGGAACTAGTCG

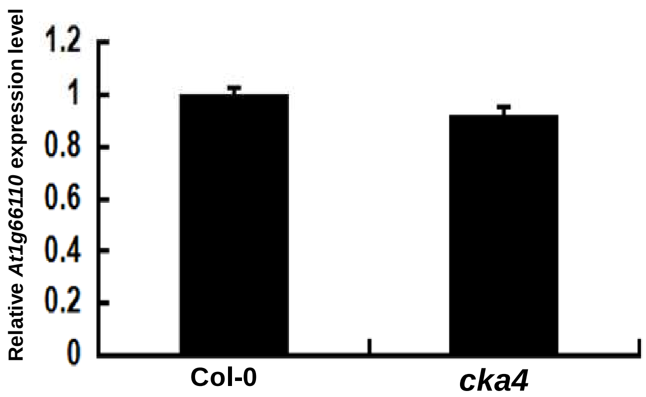

Supplementary Fig. S1. The expression level of *At1g66110* gene in Col-0 and *cka4* mutant (the progeny of CS311135).

Primers were designed downstream of the T-DNA-insertion site of *At1g66110* to identify the elimination of the At1g66110 mutation in the progeny of CS311135. The result showed that a similar transcript level appeared in Col-0 and *cka4* plants. Relative expression level is normalized to Col-0 and values are mean ± SD of three biological replicates.

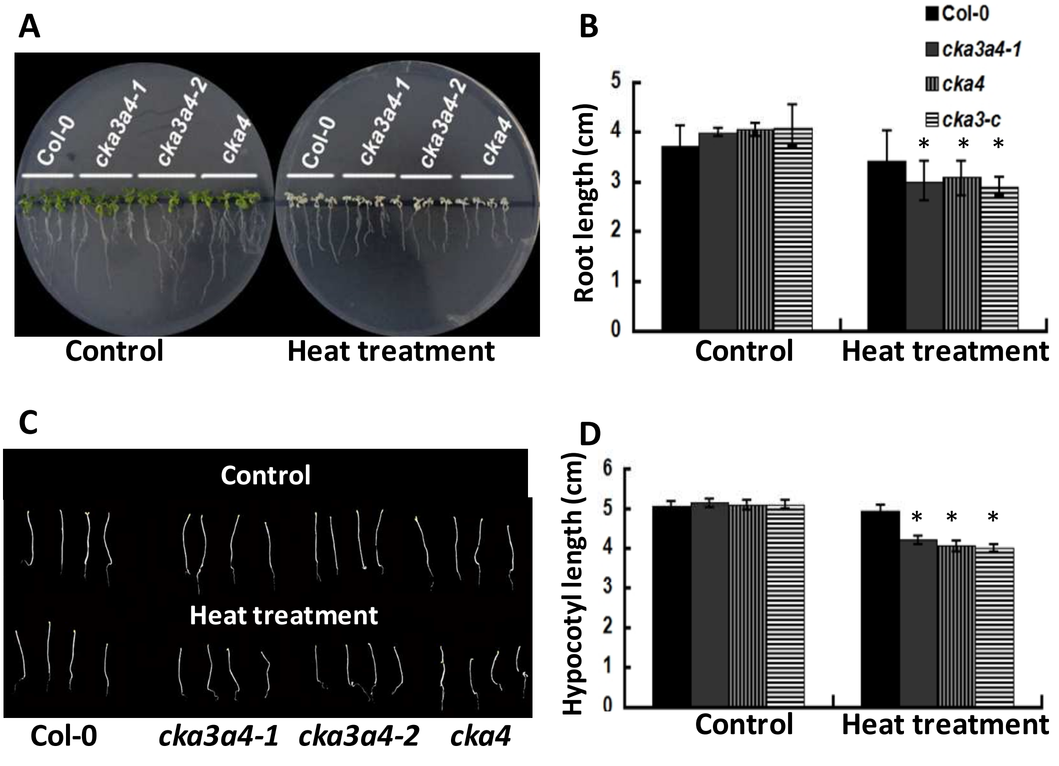

Supplementary Fig. S2. Root and hypocotyl elongation in Col-0, *cka4* and *cka3 cka4* mutants after heat treatment.

(**A**, **B**) Root length. Five-day-old seedlings were subjected to the heat stress regime (37°C for 2h, 22°C or 2h and 45°C for 2.5 h), followed by 5-day recovery at 22°C.

(**C**, **D**) Hypocotyl length. The 2.5-day-old dark-grown seedlings were exposed to the heat treatment regime, followed by 5-day recovery at 22°C in dark.

Values are mean ± SD of 200 seedlings. Asterisks indicate significant differences between mutant and Col-0 plants (**P* < 0.05).

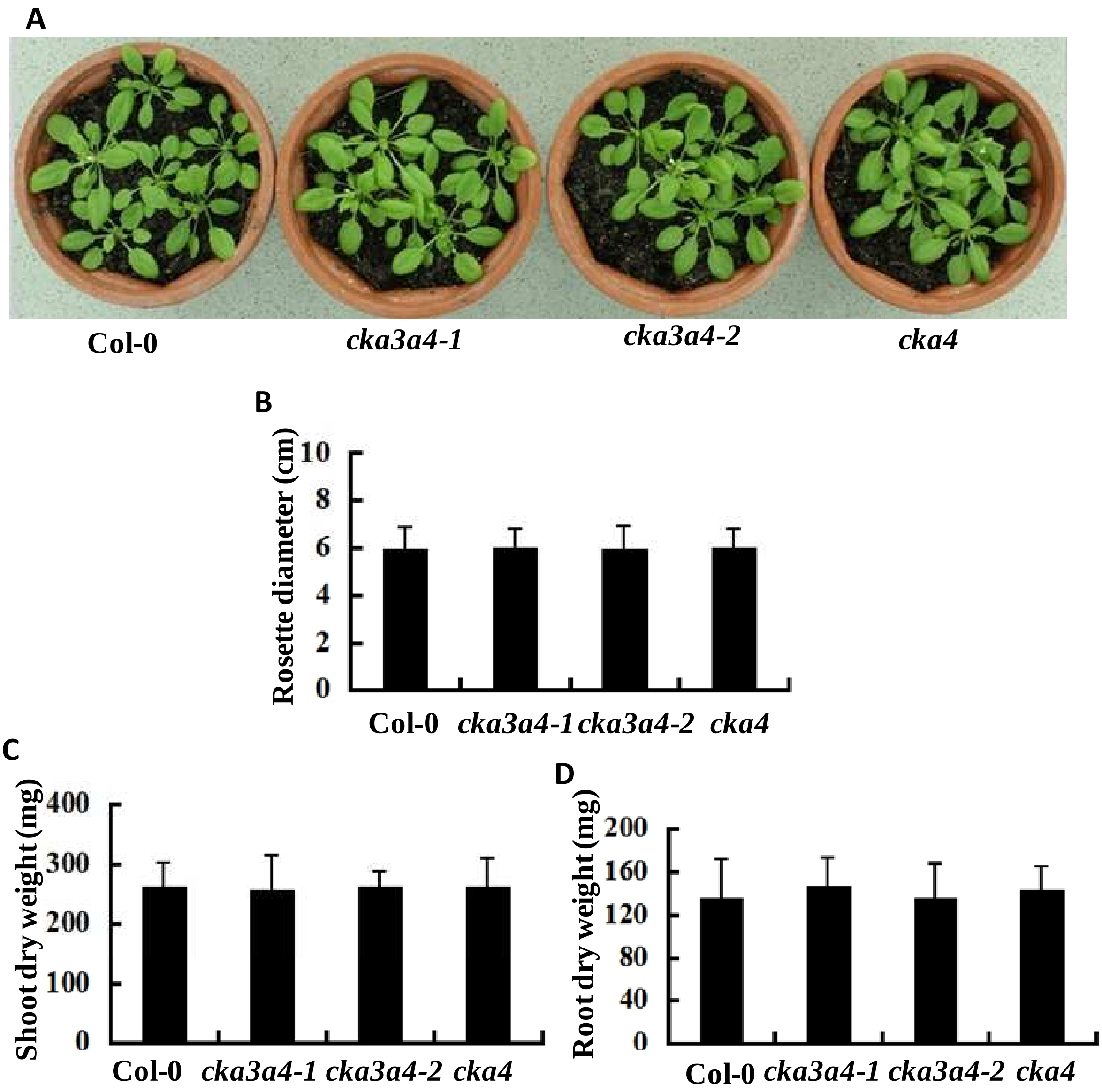

Supplementary Fig. S3 . Comparative analysis of growth parameters of wild-type Col-0, *cka4* and *cka3 cka4* mutant plants.
(**A**) Four-week-old wild-type and three mutant plants; (**B**) rosette diameters; (**C**) shoot dry weight (mg per plant) and (**D**) root dry weight (mg per plant).
Three-week-old soil-grown plants were harvested for comparative analyses of biomass production and rosette sizes. For measurement of dry weight samples were heated at 105°C for 10 min and then were dried at 65°C for 2 days. Values are means ± SD of 150 Plants. No statistically significant differences were observed between Col-0 and mutants.

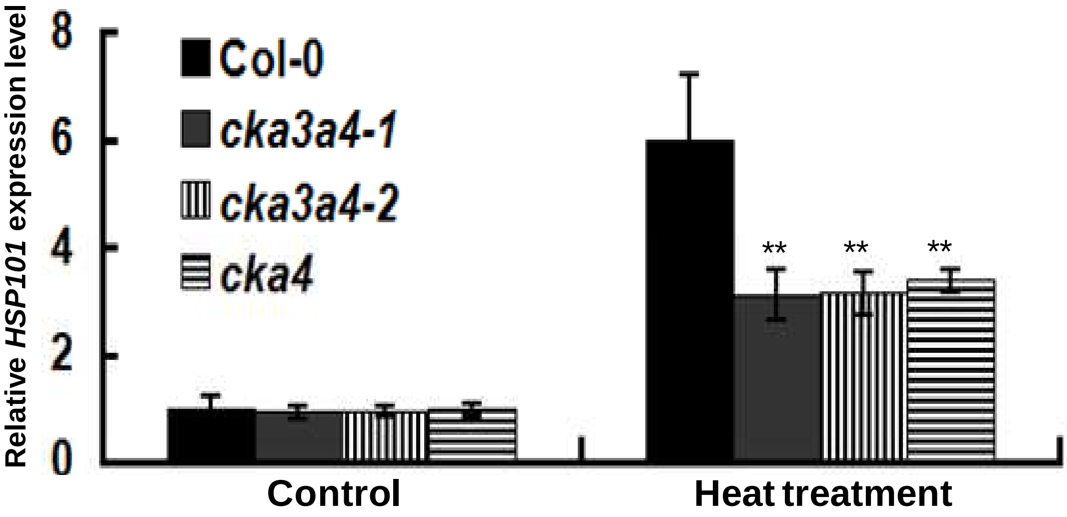

Supplementary Fig. S4. The expression level of *HSP101* at the 24 h recovery phase after a heat treatment regime.

Expression levels of *HSP101* were determined in the leaves of 30-day-old Col-0, *cka4* and *cka3a4* mutants. The results showed that the transcript level of *HSP101* remained elevated at 24 h post heat treatment and the post–heat *HSP101* transcript level was lower in the *cka4* and *cka3a4* double mutants than Col-0. Relative expression level is normalized to Col-0 and values are mean ± SD of three biological replicates. Asterisks indicate significant differences between mutant and Col-0 (P < 0.01 using Student's *t*-test). *Cka3a4-1*, *cka3-cka4-1*; *cka3a4-2*, *cka3-cka4-2*.
